# Blood levels of D-amino acid oxidase vs. D-amino acids in reflecting cognitive aging

**DOI:** 10.1038/s41598-017-13951-7

**Published:** 2017-11-01

**Authors:** Chieh-Hsin Lin, Hui-Ting Yang, Chih-Chiang Chiu, Hsien-Yuan Lane

**Affiliations:** 1 Department of Psychiatry, Kaohsiung Chang Gung Memorial Hospital, Chang Gung University College of Medicine, Kaohsiung, Taiwan; 2 Graduate Institute of Biomedical Sciences, China Medical University, Taichung, Taiwan; 3 Center for General Education, Cheng Shiu University, Kaohsiung, Taiwan; 4 Department of Nutrition, China Medical University, Taichung, Taiwan; 5 Department of Psychiatry, Taipei City Psychiatric Center, Taipei, Taiwan; 6 Department of Psychiatry, China Medical University Hospital, Taichung, Taiwan

## Abstract

Feasible peripheral biomarker for Alzheimer’s disease (AD) is lacking. Dysregulation of N-methyl-D-aspartate (NMDA) receptor is implicated in the pathogenesis of AD. D-amino acid oxidase (DAO) and amino acids can regulate the NMDA receptor function. This study aimed to examine whether peripheral DAO and amino acids levels are characteristic of age-related cognitive decline. We enrolled 397 individuals (including amnestic mild cognitive impairment (MCI), mild AD, moderate to severe AD, and healthy elderly). DAO levels in the serum were measured using ELISA. Amino acids levels in serum were measured by high performance liquid chromatography. Severity of the cognitive deficits in subjects was assessed using Clinical Dementia Rating Scale (CDR). The DAO levels increased with the severity of the cognitive deficits. DAO levels were significantly associated with D-glutamate and D-serine levels. The Receiver Operating Characteristics analysis of DAO levels for AD patients vs. healthy controls determined the optimal cutoff value, 30.10, with high sensitivity (0.842) and specificity (0.889) (area under curve = 0.928). This is the first study indicating that the peripheral DAO levels may increase with age-related cognitive decline. The finding supports the hypofunction of NMDA receptor hypothesis in AD. Whether DAO could serve as a potential surrogate biomarker needs further studies.

## Introduction

Dementia is gaining increasing attention because of the high morbidity and mortality it causes in the population. Although cognitive deterioration is common in old age, the relationship between aging and degenerative dementia such as Alzheimer’s disease (AD) remains unclear. Whereas aging is a risk factor for AD, AD may not be an inevitable part of the aging process. The pathological changes that are detected in the brains of patients with AD, such as the presence of amyloid plaques and neurofibrillary tangles, are considered to appear several years before the development of clinical symptoms^1^. Therefore, early detection and treatment can help to prevent or slow the progression of AD.

Several mechanisms have been reported to be implicated in the pathogenesis of AD, one of them is dysregulation of glutamate neurotransmission. Glutamate plays a critical role in regulating neurogenesis, neurite outgrowth and synaptogenesis, neuronal survival, and synaptic plasticity^2^, and its signaling also underlies complex neuronal activities such as learning and memory^3^. The N-methyl-D-aspartate (NMDA) receptor, a subtype of ionotropic glutamate receptor, plays an important role in synaptic plasticity, learning, memory, and cognition^4^. In AD patients, glutamate levels were diminished in the cerebrospinal fluid (CSF)^5^ and in ante mortem brain tissue^6^, the number of glutamate terminals in the hippocampus were decreased^7^, and low levels of D-serine (an endogenous full agonist of the glycine site of NMDA receptor) and high levels of L-serine were observed in the serum^8^. Another study found that D-serine levels were higher in the CSF of probable AD patients than in control subjects^9^. The density of NMDA receptor also decreases with age^10^. Therefore, dysfunction in the glutamate neurotransmission via NMDA receptor may contribute substantially to the pathophysiology of AD.

To date, the diagnosing of AD or mild cognitive impairment (MCI, a term to describe a slight impairment in cognitive function that is accompanied with mostly normal function for daily activities)^11^ relies mainly on clinical manifestations. Favorable laboratory tests, particularly from peripheral approach, are still lacking. Gene expressions associated with modulating NMDA receptor function may be involved in the etiology of MCI and AD. D-amino acid oxidase (DAO) is a flavoenzyme that degrades D-amino acids, mainly D-serine^12–14^. Studies indicate that aging is related with reduced D-serine levels and D-serine treatment decreases the extent of neuron death, suggesting that D-serine has neuroprotective effect against apoptosis^15^. D-serine is critical for the proliferation and neuronal differentiation of neural stem cells^16^. A body of evidence suggests that DAO also plays a key role in the process of oxidative stress^17,18^.

Inhibiting the activity of DAO is one of the avenues to enhance NMDA activation. Our previous study demonstrated that sodium benzoate, a DAO inhibitor, is better than placebo in improving the cognitive function of patients with MCI or mild AD in a randomized, double-blind, placebo-controlled trial^19^. The aforementioned evidences suggest that DAO and its regulatory function on NMDA receptor may play important roles in the process of aging and its related cognitive decline.

It is difficult to collect sample from brain tissue. Thus, developing accessible peripheral biomarkers becomes more important for mental illness^20^. Peripheral gene expression may be a useful surrogate for gene expression in the CNS when the relevant gene is expressed in both^21^. We hypothesize that the level of DAO and related amino acids (including L-glutamate, D-glutamate, L-serine, D-serine, L-alanine, D-alanine, and glycine) in the peripheral blood may be associated with age-related cognitive decline. This study aims to examine whether DAO was over-expressed in people with age-related cognitive deficits, and could serve as a surrogate diagnostic biomarker.

## Results

A total of 397 subjects were enrolled: 116 healthy controls, 77 patients with amnestic MCI, 128 patients with mild AD, and 76 patients with moderate to severe AD. There were significantly more male subjects in the healthy control group than the other three groups (p = 0.027). There were significant differences in age distribution and educational levels among the four groups (p < 0.001). The percentage of subjects using anti-dementia agents (including AChEI and memantine) were also significantly different among the amnestic MCI, mild AD, and moderate-severe AD groups (p = 0.001). Among the amino acids that we measured, there were significant inter-groups differences for L-glutamate level, D-glutamate level, L-serine level, D-serine level and D to L-glutamate ratio (p = 0.029, <0.001, 0.049, 0.012, 0.002, respectively). The demographic data of the four groups are summarized in Table 1.

**Table 1:** Demographic characteristics of the overall cohort (n = 397).

	Healthy elderly (n = 116)	MCI (n = 77)	Mild AD (n = 128)	Moderate to severe AD (n = 76)	*p* Value
Demographics					
Gender, female, n (%)	54 (46.6)	45 (58.4)	80 (62.5)	50 (65.8)	0.027^a^
Age, year, mean (SD)	67.5 (9.7)	68.0 (7.5)	73.7 (8.1)	78.2 (8.7)	<0.001^b^
CDR, mean (SD)	0.0 (0.0)	0.5 (0.0)	1.0 (0.0)	2.4 (0.5)	<0.001^b^
MMSE, mean (SD)	28.1 (1.8)	23.2 (3.2)	18.5 (4.4)	10.4 (4.6)	<0.001^b^
Education, year, mean (SD)	11.2 (4.1)	6.7 (5.1)	5.3 (4.2)	5.3 (5.2)	<0.001^b^
No. of subjects using anti-dementia drugs					
Total number (%)	NA	9 (11.7)	42 (32.8)	14 (18.4)	0.001^a*^
Donepezil (dose, mean ± SD)	NA	8 (6.9 ± 2.6)	25 (9.0 ± 2.0)	6 (10.0 ± 0.0)	0.039^a*^
Rivastigmine (dose, mean ± SD)	NA	1 (9.0)	8 (7.3 ± 2.3)	5 (7.6 ± 1.9)	0.218^a*^
Galantamine (dose, mean ± SD)	NA	0	9 (15.1 ± 2.7)	1 (16.0)	0.015^a*^
Memantine (dose, mean ± SD)	NA	0	0	2 (20.0 ± 0.0)	0.066^a*^
DAO level (ng/mL), mean (SD)	23.9 (11.2)	32.2 (10.8)	38.1 (14.4)	41.4 (19.5)	<0.001^b^
DAO level (ng/mL) in subjects using anti-dementia drugs, mean (SD)	NA	33.1 (12.0)	37.0 (11.8)	43.3 (18.7)	0.179^b*^
DAO level (ng/mL) in subjects without anti-dementia drugs, mean (SD)	23.9 (11.2)	32.1 (10.7)	38.7 (15.6)	41.0 (19.8)	<0.001^b^
Glycine level (ng/mL), mean (SD)	3815.9 (1333.0)	4198.5 (1351.1)	4717.4 (2086.8)	4208.2 (1238.7)	0.080^b^
L-glutamate level (ng/mL), mean (SD)	7057.1 (3915.1)	12317.1 (7622.4)	9377.5 (6733.1)	8549.7 (5551.8)	0.027^b^
D-glutamate level (ng/mL), mean (SD)	1620.4 (558.2)	1097.8 (284.0)	1031.9 (775.8)	598.3 (551.9)	<0.001^b^
L-serine level (ng/mL), mean (SD)	2858.3 (790.7)	3601.8 (1537.5)	3475.6 (1140.5)	3195.3 (1141.2)	0.049^b^
D-serine level (ng/mL), mean (SD)	30.8 (11.6)	44.6 (25.8)	47.5 (29.6)	50.1 (25.5)	0.012^b^
L-alanine level (ng/mL), mean (SD)	11347.0 (3250.9)	11643.3 (3043.1)	12864.6 (3977.4)	12325.0 (3270.9)	0.198^b^
D-alanine level (ng/mL), mean (SD)	30.2 (38.0)	35.1 (34.2)	27.8 (34.3)	39.0 (41.2)	0.528^b^
D/L-glutamate ratio, mean (SD)	0.305 (0.221)	0.149 (0.132)	0.166 (0.182)	0.126 (0.237)	0.002^b^
D/L-serine ratio, mean (SD)	0.011 (0.005)	0.014 (0.010)	0.015 (0.010)	0.017 (0.009)	0.074^b^
D/L-alanine ratio, mean (SD)	0.003 (0.003)	0.003 (0.004)	0.002 (0.003)	0.003 (0.003)	0.435^b^

NA, not associated; ^a^Chi-square test; ^b^ANOVA test; ^c^Mann-Whitney U test; ^*^Comparison among MCI, mild AD and moderate to severe AD groups

Abbreviations: CDR, Clinical Dementia Rating; MMSE, Mini Mental Status Examination; DAO, D-amino acid oxidase.

We further matched the four groups in terms of gender, age, and education. In the matched cohort, there were no significant difference among the four groups in gender, age, and education (p = 0.957, 0.666, 0.780, respectively). Among the amnestic MCI, mild AD, and moderate-severe AD groups, there was also no significant difference in the frequence of anti-dementia agents use (p = 0.072). Among the amino acids that we measured, tIhere were significant inter-groups differences for D-glutamate level, glycine level, and D to L-glutamate ratio (p < 0.001, 0.037, 0.043, respectively) in the matched cohort. The demographic data of the matched cohort are summarized in Table 2.

**Table 2:** Demographic characteristics of the matched cohort (n = 218).

	Healthy elderly (n = 50)	MCI (n = 44)	Mild AD (n = 82)	Moderate to severe AD (n = 42)	*p* Value
Demographics					
Gender, female, n (%)	24 (48.0)	23 (52.3)	42 (51.2)	20 (47.6)	0.957^a^
Age, year, mean (SD)	71.8 (9.3)	71.6 (7.3)	72.6 (9.0)	73.7 (8.7)	0.666^b^
CDR, mean (SD)	0.0 (0.0)	0.5 (0.0)	1.0 (0.0)	2.5 (0.6)	<0.001^b^
MMSE, mean (SD)	27.5 (1.9)	23.5 (3.1)	19.0 (4.4)	10.5 (5.4)	<0.001^b^
Education, year, mean (SD)	8.0 (3.5)	7.6 (5.0)	7.7 (3.2)	7.1 (5.5)	0.780^b^
No. of subjects using anti-dementia drugs					
Total number (%)	NA	8 (18.2)	28 (34.1)	8 (19.0)	0.072^a*^
Donepezil (dose, mean ± SD)	NA	7 (6.4 ± 2.4)	14 (8.6 ± 2.3)	4 (10.0 ± 0.0)	0.522^a*^
Rivastigmine (dose, mean ± SD)	NA	1 (9.0)	7 (7.1 ± 2.4)	3 (6.7 ± 2.1)	0.393^a*^
Galantamine (dose, mean ± SD)	NA	0	7 (14.9 ± 3.0)	0	0.022^a*^
Memantine (dose, mean ± SD)	NA	0	0	1 (20.0)	0.221^a*^
DAO level (ng/mL), mean (SD)	24.1 (11.3)	32.7 (11.6)	37.0 (14.0)	43.5 (19.3)	<0.001^b^
DAO level (ng/mL) in subjects using anti-dementia drugs, mean (SD)	NA	32.1 (12.4)	35.1 (10.9)	48.8 (21.0)	0.028^b*^
DAO level (ng/mL) in subjects without anti-dementia drugs, mean (SD)	24.1 (11.3)	32.8 (11.6)	38.0 (15.3)	42.3 (19.0)	<0.001^b^
Glycine level (ng/mL), mean (SD)	3951.4 (1506.2)	3762.5 (1058.4)	4887.0 (1870.1)	4101.6 (1023.1)	0.037^b^
L-glutamate level (ng/mL), mean (SD)	6313.4 (1805.5)	11821.5 (8143.5)	9716.8 (7167.3)	8660.2 (5988.1)	0.113^b^
D-glutamate level (ng/mL), mean (SD)	1579.9 (528.3)	1132.4 (318.7)	955.3 (764.4)	643.4 (589.3)	<0.001^b^
L-serine level (ng/mL), mean (SD)	2874.2 (889.7)	3391.8 (1682.5)	3517.9 (1089.2)	3351.2 (1272.5)	0.286^b^
D-serine level (ng/mL), mean (SD)	29.3 (9.9)	38.4 (13.1)	47.0 (30.9)	48.0 (26.3)	0.062^b^
L-alanine level (ng/mL), mean (SD)	11725.5 (3854.0)	11587.6 (3221.2)	13678.2 (4472.4)	12149.4 (3473.8)	0.169^b^
D-alanine level (ng/mL), mean (SD)	27.7 (34.8)	44.7 (41.1)	28.6 (36.0)	21.2 (22.9)	0.320^b^
D/L-glutamate ratio, mean (SD)	0.271 (0.111)	0.147 (0.100)	0.146 (0.141)	0.136 (0.261)	0.043^b^
D/L-serine ratio, mean (SD)	0.011 (0.005)	0.014 (0.007)	0.014 (0.009)	0.016 (0.010)	0.345^b^
D/L-alanine ratio, mean (SD)	0.002 (0.003)	0.004 (0.005)	0.002 (0.002)	0.002 (0.002)	0.088^b^

NA, not associated; ^a^Chi-square test; ^b^ANOVA test; ^c^Mann-Whitney U test; ^*^Comparison among MCI, mild AD and moderate to severe AD groups.

Abbreviations: CDR, Clinical Dementia Rating; MMSE, Mini Mental Status Examination; DAO, D-amino acid oxidase.

### DAO levels were higher in patients with cognitive impairment

**Figure 1:**
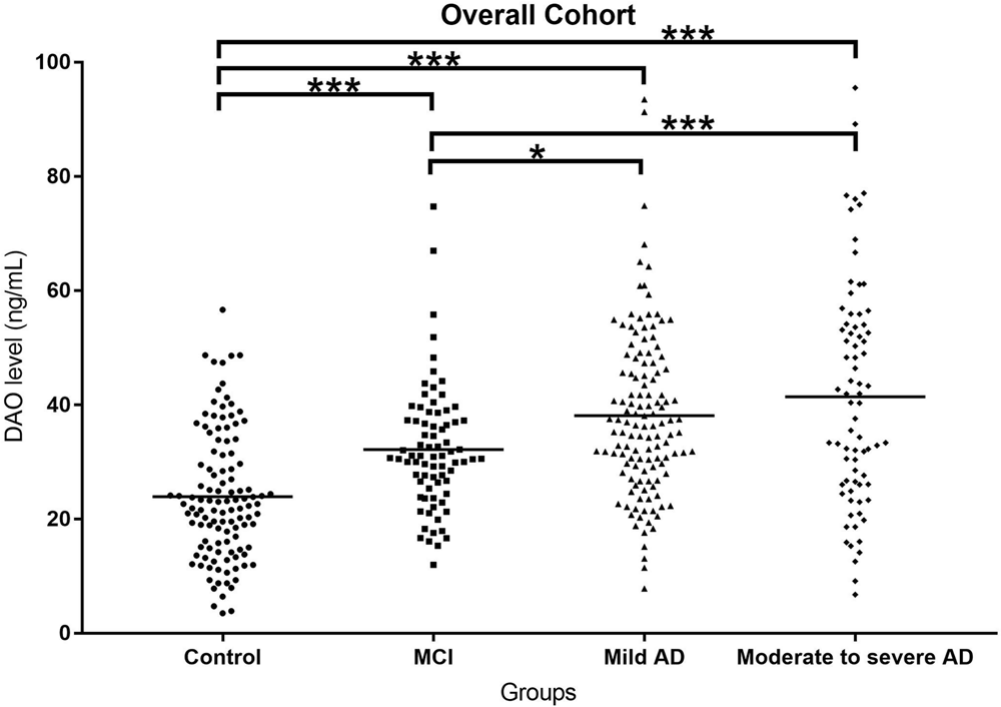
DAO, D-amino acid oxidase; MCI, mild cognitive impairment; AD, Alzheimer’s disease. *P < 0.05; ***P < 0.001.

The DAO levels in the healthy controls, amnestic MCI, mild AD, and moderate to severe AD were 23.9 ± 11.2, 32.2 ± 10.8, 38.1 ± 14.4 and 41.4 ± 19.5, respectively (p < 0.001) (Table 1 and Fig. 1). Post-hoc analysis using Bonferroni method showed that the DAO level in healthy controls was significantly lower than that of patients with amnestic MCI, mild AD and moderate to severe AD (p < 0.001, p < 0.001, p < 0.001, respectively). The DAO level in patients with amnestic MCI was significantly lower than that of patients with mild AD and moderate to severe AD (p = 0.023, p < 0.001, respectively). There was no significant difference of DAO level between patients with mild AD and patients with moderate to severe AD (p = 0.642). The DAO level was significantly correlated with CDR score (r = 0.389, p < 0.001). The DAO levels in patients using anti-dementia drugs were very similar to those without anti-dementia drugs (Table 1).

**Figure 2:**
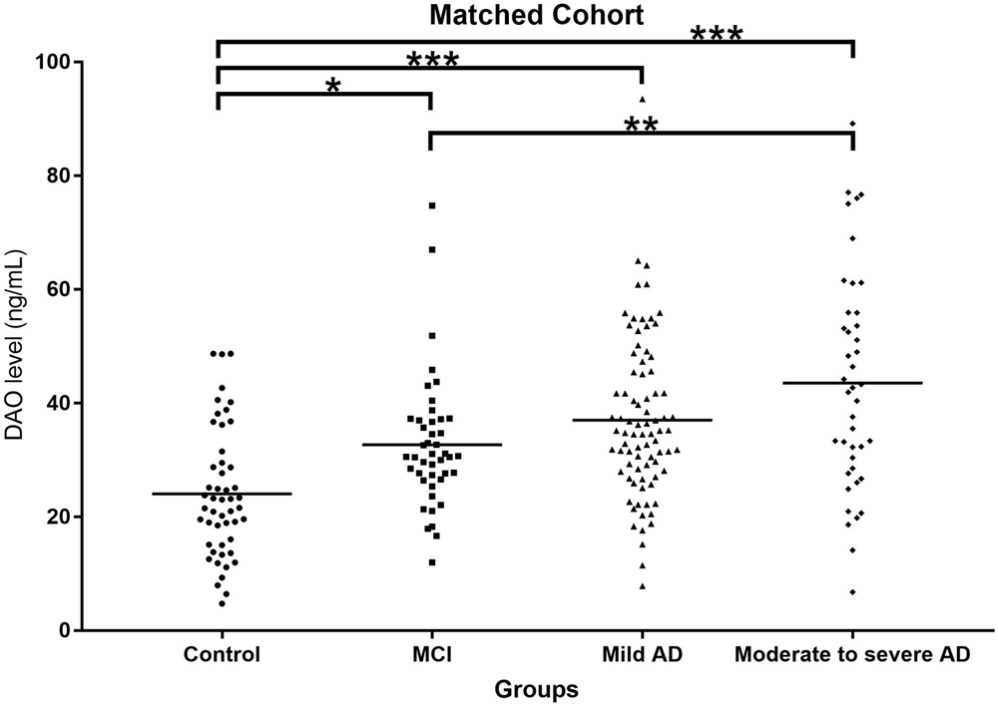
DAO, D-amino acid oxidase; MCI, mild cognitive impairment; AD, Alzheimer’s disease. *P < 0.05; **P < 0.01; ***P < 0.001.

For the matched cohort, the DAO levels in the healthy controls, amnestic MCI, mild AD and moderate to severe AD were 24.1 ± 11.3, 32.7 ± 11.6, 37.0 ± 14.0 and 43.5 ± 19.3, respectively (p < 0.001) (Table 2 and Fig. 2). Post-hoc analysis using Bonferroni method showed that the DAO level in healthy controls was significantly lower than that of patients with amnestic MCI, mild AD and moderate to severe AD (p = 0.022, p < 0.001, p < 0.001, respectively). The DAO level in patients with amnestic MCI was significantly lower than that of patients with moderate to severe AD (p = 0.003). There was no significant difference of DAO level between patients with amnestic MCI and patients with mild AD (p = 0.634). There was also no significant difference of DAO level between patient with mild AD and patients with moderate to severe AD (p = 0.096). The DAO level was also significantly correlated with CDR score in the matched cohort (r = 0.394, p < 0.001). The DAO levels in patients using anti-dementia drugs were also very similar to those without anti-dementia drugs in the matched cohort (Table 2).

### DAO level was associated with D-glutamate and D-serine levels

The relationship between DAO and amino acids was testified in multiple linear regression analyses. The regression models were adjusted with age, sex and education. Due to the high co-linearity between amino acids levels and D- to L- form ratios, only amino acids levels were entered in the regression models because amino acids levels were more significantly associated with DAO levels than D- to L- form ratios (data not shown). In the overall cohort, DAO level was significantly associated with D-glutamate level, D-serine level and L-alanine level (adjusted R square = 0.290) (Table 3). In the matched cohort, DAO level was significantly associated with D-glutamate level (p < 0.001) and D-serine level (p = 0.007) (adjusted R square = 0.346) (Table 3).

**Table 3:** Multiple linear regression analyses of independent factors associated with DAO level in overall and matched cohorts (stepwise).

Variable	B (SE)	t	*P*
Overall cohort (N = 146)			
D-glutamate level (ng/mL)	−0.010 (0.002)	−5.159	<0.001
D-serine level (ng/mL)	0.147 (0.044)	3.327	0.001
L-alanine level (ng/mL)	0.001 (0.000)	2.338	0.021
Adjusted R square = 0.290			
**Matched cohort (N = 87)**			
D-glutamate level (ng/mL)	−0.013 (0.002)	−5.684	<0.001
D-serine level (ng/mL)	0.165 (0.060)	2.757	0.007
Adjusted R square = 0.346			

The regression model was adjusted with age, sex and education. The variables were L-glutamate level, D-glutamate level, L-serine level, D-serine level, glycine level, L-alanine level, and D-alanine level. Significant variables are shown in the Table (p < 0.05).

### Factors associated with cognitive function

#### CDR Score

Multiple linear regression analyses were used to test the potential factors associated with CDR score. The regression models were adjusted with age, sex and education. Similarly, D- to L- forms amino acids ratios were not entered in the regression model due to high co-linearity with amino acids levels. In the overall cohort, CDR score was significantly associated with age, education, DAO level, D-glutamate level and D-serine level (adjusted R square = 0.420) (Table 4). In the matched cohort, CDR was significantly associated with DAO level (p = 0.011), D-glutamate level (p = 0.001) and D-serine level (p = 0.005) (adjusted R square = 0.395) (Table 4).

**Table 4:** Multiple linear regression analyses of independent factors associated with Clinical Dementia Rating (CDR) scale in overall and matched cohort (stepwise).

Variable	B (SE)	t	*P*
Overall cohort (N = 146)			
Age (year)	0.016 (0.006)	2.620	0.010
Education (year)	−0.043 (0.015)	−2.883	0.005
DAO level (ng/mL)	0.013 (0.004)	2.967	0.004
D-glutamate level (ng/mL)	0.000 (0.000)	−3.882	<0.001
D-serine level (ng/mL)	0.005 (0.002)	2.042	0.043
Adjusted R square = 0.420			
**Matched cohort (N = 87)**			
DAO level (ng/mL)	0.016 (0.006)	2.615	0.011
D-glutamate level (ng/mL)	0.000 (0.000)	−3.348	0.001
D-serine level (ng/mL)	0.010 (0.003)	2.910	0.005
Adjusted R square = 0.395			

The regression model was adjusted with age, sex and education. The variables were L-glutamate level, D-glutamate level, L-serine level, D-serine level, Glycine level, L-alanine level, and D-alanine level. Significant variables are shown in the Table (p < 0.05).

#### MMSE Score

Multiple linear regression analyses were used to test the potential factors associated with MMSE score. The regression models were adjusted with age, sex and education. Similarly, D- to L- forms amino acids ratios were not entered in the regression model due to high co-linearity with amino acids levels. In the overall cohort, MMSE score was significantly associated with education, DAO level and D-glutamate level (adjusted R square = 0.344) (Supplementary Table 1). In the matched cohort, MMSE was significantly associated with DAO level (p < 0.001) (adjusted R square = 0.253) (Supplementary Table 1).

### DAO, D-glutamate and D-serine levels differentiated between patients with AD and healthy controls

ROC analysis was applied to determine the cutoff value of DAO level and amino acids as potential predictors for AD by plotting the proportion of true-positive results (sensitivity) vs. the proportion of false-positive results (1 - specificity). Patients with amnestic MCI were excluded for this analysis because amnestic MCI has not yet been confirmed as a definite diagnosis. In the overall cohort, the ROC analysis revealed that DAO level for all AD patients vs. healthy controls determined an optimal cutoff value, 29.74, with a good sensitivity (0.823) and modest specificity (0.767) (AUC = 0.868). D-glutamate had the highest AUC (0.798) among all amino acids with an excellent sensitivity (0.967) and modest specificity (0.531) at the optimal cutoff value 975.04. D/L-glutamate ratio had similar AUC (0.784) with a good sensitivity (0.833) and modest specificity (0.708) at the optimal cutoff value 0.18. D-serine level also had good AUC (0.740) with a modest sensitivity (0.635) and good specificity (0.800) at the optimal cutoff value 35.85. We further combined DAO and amino acid levels to generate an equation by logistic regression model. The combination of DAO, D-glutamate and D-serine had good AUC (0.832) with a modest sensitivity (0.645) and excellent specificity (0.967) at the optimal cutoff value 37.00 (Table 5).

**Table 5:** ROC curve analysis of predictive factors for Alzheimer’s disease vs. healthy controls in overall and matched cohort

Predictive factors	Cut-off	Sensitivity	Specificity	AUC	*P*
Overall cohort (N = 126)					
DAO level (ng/mL)	29.74	0.823	0.767	0.868	<0.001
D-glutamate level (ng/mL)	975.04	0.967	0.531	0.798	<0.001
D/L-glutamate ratio	0.18	0.833	0.708	0.784	<0.001
D-serine level (ng/mL)	35.85	0.635	0.800	0.740	<0.001
DAO/D-glutamate/D-serine*	37.00	0.645	0.967	0.832	<0.001
*(DAO × 1.158) − (D-glutamate × 0.999) + (D-serine × 1.041). The equation was generated by logistic regression model.					
**Matched cohort (N = 75)**					
DAO level (ng/mL)	30.10	0.842	0.889	0.928	<0.001
D-glutamate level (ng/mL)	1054.24	0.889	0.632	0.813	<0.001
D/L-glutamate ratio	0.18	0.889	0.737	0.834	<0.001
D-serine level (ng/mL)	39.19	0.544	0.833	0.739	0.002
DAO/D-serine*	80.18	0.783	1.000	0.940	<0.001

*(DAO × 1.278) + (D-serine × 1.089). The equation was generated by logistic regression model.

Patients with mild cognitive impairment (CDR = 0.5) were excluded.

For the matched cohort, the ROC analysis revealed that DAO level for all AD patients vs. healthy controls determined an optimal cutoff value, 30.10, with a good sensitivity (0.842) and good specificity (0.889) (AUC = 0.928). D/L-glutamate ratio had the highest AUC (0.834) among all amino acids with a good sensitivity (0.889) and modest specificity (0.737) at the optimal cutoff value 0.18. D-glutamate level had similar AUC (0.813) with a good sensitivity (0.889) and modest specificity (0.632) at the optimal cutoff value 1054.24. D-serine level also had good AUC (0.739) with a modest sensitivity (0.544) and good specificity (0.833) at the optimal cutoff value 39.19. We also combined DAO and amino acid levels to generate an equation by logistic regression model. The combination of DAO and D-serine had excellent AUC (0.940) with a modest sensitivity (0.783) and excellent specificity (1.000) at the optimal cutoff value 80.18 (Table 5).

## Discussion

To our knowledge, the current study is the first one demonstrating that the DAO levels in peripheral blood are higher in patients with MCI and AD than healthy individuals, and the peripheral DAO level is positively correlated with the severity of cognitive impairment. DAO can regulate the function of NMDA receptor via metabolizing D-amino acids, particularly D-serine. Our previous clinical trial using a DAO inhibitor, sodium benzoate, has shown beneficial effect for patients with early-phase AD^19^. The findings of higher DAO levels in patients with MCI and AD are in line with the attenuated NMDA receptor function hypothesis in the aging process and related cognitive decline^10^. More importantly, developing potential biomarker for cognitive aging from accessible peripheral blood is much more feasible than collecting samples from brain tissue or CSF, which makes early detection and prevention easier.

The findings from peripheral blood in our study support the previous study that D-serine levels were higher in the CSF of AD patients than in control subjects^9^. Treating neurons with both NMDA and D-serine produced an additive effect than D-serine alone in suppressing neuronal death^15^. In a 6-wk double-blind, placebo-controlled, crossover trial conducted on Parkinson’s disease patients, treatment with a D-serine adjuvant was shown to reduce the total scores of Unified Parkinson’s Disease Rating Scale, Simpson-Angus Scale for Extrapyramidal Symptoms, and Positive and Negative Syndrome Scale (PANSS)^22^. An earlier study found that D-serine level declines and DAO increases in the cerebellum of rats during early postnatal development^23^. A recent study found that the *DAO* mRNA expression levels were higher in the cerebellum compared to other brain regions, and the *DAO* mRNA levels were positively correlated with age less than 2 years in the cerebellum and amygdala in normal human post-mortem brain samples^24^. The above *in vitro* and *in vivo* studies suggest that neurotransmission via NMDA receptor is pivotal for regulating the cognitive function in the aging brain.

D-glutamate level was found to decrease with the severity of cognitive impairment in this study. D-glutamate can be detected in blood^25^, saliva^26^ and urine^27^. D-glutamate has been detected in several brain areas, peripheral tissues, plasma and urine of rodents by Hamase’s group^28,29^. The D-glutamate level and other amino acid levels in this study were similar to those in a previous study in human plasma^30^. Mangas *et al*. found that the D-glutamate levels were increased in several brain regions that are important for cognitive and behavioral regulations in rats^31^. Although L-glutamate is the most abundant excitatory neurotransmitter in the brain^32^, D-glutamate level is relatively higher in the cortex than other brain regions^33^, suggesting that D-glutamate may be important for high cortical functions. It is noteworthy to investigate the role of D-glutamate in aging in future studies.

Our finding of increased DAO and D-serine levels as well as decreased D-glutamate levels for people with cognitive aging in the matched cohort were very similar to that of the overall cohort. The findings suggest that the changes of DAO, D-glutamate and D-serine with cognitive deterioration are not affected by the demographic characterics including age, sex and education. This observation supports the viewpoint that cognitive decline may be not necessarily an inevitable part of the normal aging process. However, whether DAO and amino acids levels in patients with cognitive impairment really progress with aging needs further elucidation by prospective study.

Our study has several limitations. First, since this is a cross-sectional study, whether the findings keep constant over cognitive deteriorating process requires further prospective, longitudinal study. Second, although a previous GWAS study has found a suggestive evidence of association for the D-serine plasma-CSF ratio at the *DAO* gene from the general population^34^, the peripheral blood-CNS relationship of DAO and amino acids expressions also needs to be examined by studies among individuals with AD. Third, the amino acids levels were not measured for all participants in this study due to limited amounts of blood samples. However, the average sample size with amino acids levels in each group for the matched cohort is around 22, making the results still of some value. Fourth, our findings need to be testified in various racial populations by other groups. Fifth, D-aspartate level was not assayed in this study. D-aspartate can bind to the glutamate site of GluN2 receptor to enhance the NMDA receptor function^35^. Animal studies found that D-aspartate enhanced NMDA receptor-dependent long-term potentiation in the hippocampus^36^, and rescued the synaptic plasticity decay in the hippocampus of aged mice^35^. The role of D-aspartate in cognitive aging deserves further investigation. Lastly, other neurodegenerative diseases (e.g. amyotrophic lateral sclerosis and schizophrenia) that are associated with altered DAO level^37,38^ could not be entirely ruled out although we had excluded subjects with obvious brain or mental disorders by history, physical examinations and laboratory assessments.

Taken together, peripheral DAO levels are higher in patients with cognitive decline. The findings support the hypofunction of NMDA receptor-mediated neurotransmission hypothesis in MCI and AD^39^. Since AD is a complex and multifactorial disease, it is reasonable to combine different potential biomarkers for assisting the diagnosis. For example, combining DAO level with other potential tools, e.g. amino acids levels and β-amyloid^40^, for ascertainment might be a possible approach. In the future, the potential relationship of DAO levels and amino acids levels with treatment response for AD or a subpopulation of AD) requires clarification from larger samples of patients with different severities of cognitive deficits and under various treatments.

## Methods

### Participants

All subjects were screened and recruited from the following institutes: Department of Psychiatry of Kaohsiung Chang Gung Memorial Hospital, which is a major medical center in southern Taiwan, Department of Psychiatry of China Medical University Hospital, which is a major medical center in central Taiwan, and Department of Psychiatry of Taipei City Municipal Hospital, which is a major medical center in northern Taiwan. This study was approved by the institutional review boards of the aforementioned institutes, and carried out in accordance with the Declaration of Helsinki. After thorough description of the study to the subjects, written informed consents were obtained according to the IRB’s guidelines.

All subjects were Han Chinese, aged 50–100 years, who were physically healthy and had normal laboratory assessments (including blood routine and biochemical tests). Both patients and healthy controls were evaluated by research psychiatrists after a thorough medical workup. Patients were enrolled into this study if they [1] satisfied NINCDS-ADRDA (National Institute of Neurological and Communicative Disorders and Stroke and the Alzheimer’s Disease and Related Disorders Association)^41^ criteria for probable AD and had a Clinical Dementia Rating (CDR)^42^ score of 1, or criteria for amnestic MCI^43^ of a presumably degenerative nature defined as subjective memory complaint corroborated by an informant and insufficient global cognitive and functional impairment to meet NINCDS-ADRDA criteria and had a CDR score of 0.5, [2] had sufficient education to communicate effectively and were capable of completing the assessments of the study, and [3] agreed to participate in the study and provided informed consent. Exclusion criteria included history of significant cerebrovascular disease; Hachinski Ischemic Score >4; major neurological, psychiatric or medical conditions other than AD; substance (including alcohol) abuse or dependence; delusion, hallucination or delirium symptoms; severe visual or hearing loss; and inability to follow protocol.

There was no any Axis I or II psychiatric disorder for the healthy volunteers. All participants had no DSM-IV diagnosis of substance (including alcohol) abuse or dependence to exclude potential confounding effects. Patients with amnestic MCI had a CDR score of 0.5. Patients with mild AD had a CDR score of 1 and patients with moderate to severe AD had a CDR score 2 or greater.

Both anti-dementia drugs free and medicated patients with AD were recruited for examining possible drug effects on the DAO level. Drug-free AD patients had not taken any anti-dementia drug for at least three months. For patients who had already been on anti-dementia drugs therapy, anti-dementia drugs had to be continued for at least three months with unchanged dose before enrollment. Psychotropic use history was ascertained by interviewing the patients and their family members or care givers, contacting other health care providers and reviewing medical records. Healthy individuals had no history of exposure to anti-dementia drugs. All patients with AD were recruited from the outpatient clinic at the aforementioned institutes, and all healthy volunteers were from the communities in northern, central and southern Taiwan.

Among 281 patients with amnestic MCI or AD, 216 were anti-dementia drug free for 3 months or longer and the other 65 were stabilized on anti-dementia drugs (39 donepezil, 14 rivastigmine, 10 galantamine, 2 memantine) for at least 3 months. Among the drug-free patients, 206 patients were drug-naïve and the other 10 were nondrug-naïve.

### Cognitive Function Assessments

The participant’s cognitive function was assessed by CDR and Mini-Mental State Examination (MMSE)^44^. MMSE is a commonly used tool for the measurement of cognitive impairment and the screening for dementia in the elderly people^44^. However, one of the disadvantages to the utilization of the MMSE is that it is affected by age and education^45^. Another disadvantage of the MMSE is its lower level of sensitivity for mild degrees of impairment^46^.

On the other hand, CDR exhibits excellent discriminatory ability in the very mild stages of dementia^47^. Besides, studies have shown that the CDR appears to be a reliable and valid tool for assessing and staging dementia with moderate to high overall inter-rater reliability^48^. Therefore, the grouping of subjects and further analysis with blood parameters in this study were based on CDR scores, which can reflect the global cognitive impairment of the subjects and not be affected by age and education level^49^.

### Laboratory Assessments

For both patients and healthy controls, blood sampling was done during 8–12AM after fasting for more than eight hours. Ten ml of blood was collected by personnel trained in phlebotomy using sterile technique. The blood specimens were processed immediately by centrifugation at 1000 x g. After centrifugation, serum was quickly dissected, immediately stored at −80 °C until further measurement.

### DAO level measurement

DAO protein concentrations were measured using commercially available enzyme-linked immunosorbent assay (ELISA) kits according to the manufacture’s recommended protocol (Cloud-Clone Corp, Houston, TX, USA). Briefly, 100 μL serum samples and the standard were added to each well of a 96-well plate. The solutions were incubated for 1 hour at 37 °C. The liquid was then removed. 100 μL Detection Reagent A was added to each well and incubated for 1 hour at 37 °C. Each well was washed for 3 times. 100 μL of Detection Reagent B was added to each well, and the solutions were incubated for 30 minutes at 37 °C. Each well was washed for 5 times and then incubated with 90 μL substrate solution for 20 min at 37 °C with the protection from light. 50 μL stop solution was added to each well. A Benchmark Plus Microplate Reader (Bio-Rad) was used to read the optical density at 450 nm. The concentrations of DAO in the samples were determined according to a standard curve.

According the instruction manual of the DAO ELISA kit, the assay has high sensitivity (the minimum detectable dose of DAO is typically less than 0.56 ng/mL) and excellent specificity for detection of DAO. To the best of current skills and knowledge, no significant cross-reactivity or interference between DAO and analogues was observed.

### Amino acids levels measurement

Serum was firstly extracted by methanol (1:3, by volume), then filtered after 15 min centrifugation (1500 × g) with nylon membranes (0.45 mM, Minisart SRP4, Sartorius, Germany). The filtrate was diluted with proper amount of 20% methanol then derivatized with N-isobutyl-L-cycteine (IBC) and O-phthaldialdehyde mixture for 5 minutes then injected into high performance liquid chromatography (HPLC, L-7100 Pump, L7250 Autosampler, L-7250, with L7480 flourscence Detector, Hitachi, Japan) for analysis. Analytical column (Grom-Sil OPA-2, 5 µm, 250 mm * 4 mm, Part No: GSOP 20512S2504, SAP No: 5113679, Grace, US) with guard column (Grom-Sil OPA-2, 5 µm, 10 mm × 4 mm, Part No: GSOP20512v0104V, Grace, US) were used for the determination. Isocratic elution of mobile phase A (23 mM sodium acetate, pH 6.0) and B (50 mL acetonitrile in 600 mL methanol) were performed under fluorescence detection (excitation 260 nm, emission 455 nm), respectively. Retention time of each amino acid was L-glutamine, 25.5 min; D-glutamine, 27.3 min; L-serine, 33.6 min; D-serine, 35.8 min; glycine, 41.5 min; L-alanine, 47.2 min; D-alanine, 50.3 min, respectively. All amino acids levels were double-checked by performing HPLC analyses for two times in order to confirm that the peaks were not artifact.

### Statistical Analysis

All subjects’ clinical characteristics, DAO levels and amino acids levels were presented as mean ± SD or number (percentage). All statistical methods were performed using IBM SPSS Statistics version 22.0 (SPSS inc.). All mean values between groups were compared using independent t test or Mann-Whitney U test for two groups, one-way ANOVA or Kruskal-Wallis test for three groups, and percentages using χ2 test. Multiple linear regression and Receiver Operating Characteristics (ROC) analysis were used to generate predictive models and to evaluate for the significant predictors of AD patients. A P value less than 0.05 was considered statistically significant.

## Electronic supplementary material

Supplementary Table 1
