# Supplementary material for: Blood levels of D-amino acid oxidase vs. D-amino acids in reflecting cognitive aging
Source: Sci Rep. 2017 Nov 1;7:14849. doi: 10.1038/s41598-017-13951-7 (PMC5665939; doi:10.1038/s41598-017-13951-7)
Supplement: Supplementary file 1 — Supplementary Table 1 [file 41598_2017_13951_MOESM1_ESM.docx]

**Blood levels of D-amino acid oxidase vs. D-amino acids in reflecting cognitive aging**

Chieh-Hsin Lin, M.D., Ph.D. ^a, b, c^, Hui-Ting Yang, Ph.D.^d^, Chi-Chiang Chiu, M.D.^e^, Hsien-Yuan Lane, M.D., Ph.D. ^b, f,^ *

^a^ Department of Psychiatry, Kaohsiung Chang Gung Memorial Hospital, Chang Gung University College of Medicine, Kaohsiung, Taiwan

^b^ Graduate Institute of Biomedical Sciences, China Medical University, Taichung, Taiwan

^c^ Center for General Education, Cheng Shiu University, Kaohsiung, Taiwan

^d^ Department of Nutrition, China Medical University, Taichung, Taiwan

^e^ Department of Psychiatry, Taipei City Psychiatric Center, Taipei, Taiwan

^f^ Department of Psychiatry, China Medical University Hospital, Taichung, Taiwan

Running Title: Higher DAO in cognitive aging

* Corresponding author at: Department of Psychiatry, China Medical University Hospital, No. 2, Yuh-Der Road, Taichung 404, Taiwan.

*Email address:* hylane@gmail.com (H.-Y. Lane).

**Conflict of interest statement:** All authors declare that they have no conflicts of interest.

**Supplementary Table 1***.* Multiple linear regression analyses of independent factors associated with MMSE scale in overall and matched cohort (stepwise)

| Overall cohort (N=146) |  |  |  |
| --- | --- | --- | --- |
| Variable | B (SE) | t | *P* |
| Education (year) | 0.346 (0.131) | 2.641 | 0.009 |
| DAO level (ng/mL) | -0.146 (0.034) | -4.270 | <0.001 |
| D-glutamate level (ng/mL) | 0.003 (0.001) | 3.521 | 0.001 |
| Adjusted R square = 0.344 |  |  |  |
|  |  |  |  |
| Matched cohort (N=87) |  |  |  |
| Variable | B (SE) | t | *P* |
| DAO level (ng/mL) | -0.229 (0.052) | -4.400 | <0.001 |
| Adjusted R square = 0.253 |  |  |  |

The regression model was adjusted with age, sex and education. The variables were L-glutamate level, D-glutamate level, L-serine level, D-serine level, Glycine level, L-alanine level, and D-alanine level. Significant variables are shown in the Table (p<0.05).
